# Venereal Affections

**Published:** 1851-03

**Authors:** 


### ARTICLE VII.

### *Venereal Affections.*

*Gonorrhœa.*—Give acetate of potash in half drachm doses every four hours. Even where the disease has not yielded, it has been repeatedly found, that, after a few doses of copaiba, the cure has been completed, and a relapse prevented. (Mr. J. Hilton, p. 264.)

The severe forms of gonorrhœa which are so exceedingly obstinate, are owing to the inflammation spreading along the entire canal, extending over to the mucous membrane of the bladder itself, nay, even, in some cases to the ureters and kidneys; and puss discharged through the urine is an indication of it. There is no specific remedy for this form, but when pus has once appeared in the urine, the antiphlogistic treatment must be more strictly enforced; and when the inflammatory symptoms have subsided, then, and not until, specific remedies, as balsams and cubebs, may be resorted to. (Mr. W. Colles, p. 265.)

*Syphilis.*—Surround the patient with an atmosphere of mercurial vapor, in a moist state, by the following method: Place the patient on a chair, covering him with oil-cloth lined with flannel, then fumigate him with the bisulphuret of mercury, or the grey oxide, or binoxide. The patient may remain in this mercurial atmosphere for ten minutes or half an hour. Let the patient then repose in an arm chair for a short time, and let him drink a cup of warm decoction of guaiacum, sweetened with syrup of sarsaparilla. (Mr. Langston Parker, p. 261.)

*Indurated Chancre.*—M. Ricord gives the proto-ioduret of mercury, but this does not seem to agree with patients in this country, which may be attributed to climate and difference in diet. He says mercury must be persisted in three or six months after the disease has disappeared to prevent a relapse. He uses fumigations, also, much on the same plan as Mr. Langston Parker, but thinks a spirit-lamp does not give out sufficient heat. (p. 261.)

*Phagedenic Chancre.*—Iron is the remedy, its effects are most magical. It is much used by M. Ricord. The following is the best mode of giving it. Potassio-tartarate of iron, one ounce; water six ounces. Mix. Two table-spoonfuls three times a day. (p. 262.)

*Tertiary Symptoms.*—Give the iodide of potash in *large doses*, which will benefit when small ones are of no avail. (Mr. W. Acton, p. 263.)—*Ibid.*

